# UCST-Type Thermoresponsive Sol–Gel Transition Triblock Copolymer Containing Zwitterionic Polymer Blocks

**DOI:** 10.3390/gels10050288

**Published:** 2024-04-24

**Authors:** Akifumi Kawamura, Ryogo Takahashi, Takashi Miyata

**Affiliations:** 1 Department of Chemistry and Materials Engineering, Kansai University, Suita 564-8680, Osaka, Japan; 2 Organization for Research and Development of Innovative Science and Technology, Kansai University, Suita 564-8680, Osaka, Japan

**Keywords:** thermoresponsive sol–gel transition, upper critical solution temperature (UCST), zwitterionic polymer, ammonium sulfonate polymer, ammonium sulfate polymer

## Abstract

Thermoresponsive sol–gel transition polymers are of significant interest because of their fascinating biomedical applications, including as drug reservoirs for drug delivery systems and scaffolds for tissue engineering. Although extensive research has been conducted on lower critical solution temperature (LCST)-type sol–gel transition polymers, there have been few reports on upper critical solution temperature (UCST)-type sol–gel transition polymers. In this study, we designed an ABA-type triblock copolymer composed of a poly(ethylene glycol) (PEG) block and zwitterionic polymer blocks that exhibit UCST-type thermoresponsive phase transitions. A sulfobetaine (SB) monomer with both ammonium and sulfonate (–SO_3_) groups in its side chain or a sulfabetaine (SaB) monomer with both ammonium and sulfate (–OSO_3_) groups in its side chain was polymerized from both ends of the PEG block via reversible addition–fragmentation chain-transfer (RAFT) polymerization to obtain PSB-PEG-PSB and PSaB-PEG-PSaB triblock copolymers, respectively. Although an aqueous solution containing the PSB-PEG-PSB triblock copolymer showed an increase in viscosity upon cooling, it did not undergo a sol-to-gel transition. In contrast, a sol-to-gel transition was observed when a phosphate-buffered saline containing PSaB-PEG-PSaB was cooled from 80 °C to 25 °C. The PSaB blocks with –OSO_3_ groups exhibited a stronger dipole–dipole interaction than conventional SB with –SO_3_ groups, leading to intermolecular association and the formation of a gel network composed of PSaB assemblies bridged with PEG. The fascinating UCST-type thermoresponsive sol–gel transition properties of the PSaB-PEG-PSaB triblock copolymer suggest that it can provide a useful platform for designing smart biomaterials, such as drug delivery reservoirs and cell culture scaffolds.

## 1. Introduction

Stimuli-responsive hydrogels that undergo reversible sol–gel transitions in response to external chemical and/or physical stimuli are of significant interest because of their potential applications as smart soft materials, such as sensors, actuators, drug reservoirs, bioreactors, and cell culture scaffolds for regenerative medicine [1,2,3]. Stimuli-responsive sol–gel transition polymers have been designed by introducing stimuli-responsive moieties that form chemical and physical cross-links in response to external stimuli such as temperature, pH, light, and target molecules. For example, we reported photo-responsive gelation of four-armed poly(ethylene glycol) (tetra-PEG) with photo-dimerizable cinnamoyl and maleimide groups at its ends [4]. The aqueous solution containing photo-responsive tetra-PEG exhibited a sol-to-gel transition upon photo-irradiation owing to the photo-dimerization of cinnamoyl and maleimide groups at the end of tetra-PEG, resulting in the formation of a three-dimensionally cross-linked network. The target biomolecule-responsive reversible sol–gel transition can be accomplished using bioconjugated tetra-PEG [5]. Biotin-conjugated tetra-PEG underwent a phase transition from a sol state to a gel state in response to the target avidin, which forms a biomolecular complex with biotin. The addition of free biotin to the resulting hydrogel induced its dissociation to a sol state owing to a complex exchange mechanism. Among various stimuli-responsive sol–gel transition polymers, thermoresponsive sol–gel transition polymers have been the most extensively studied [6,7,8]. Kim et al. reported pioneering work on thermoresponsive sol–gel transition polymers for medical applications [6,9]. They designed diblock and triblock copolymers composed of hydrophobic and biodegradable poly(L-lactide) (PLLA) and hydrophilic PEG blocks. An aqueous solution of the block copolymer with a high polymer concentration underwent a gel-to-sol transition as the temperature increased. The thermoresponsive sol–gel transition polymers were injected into the body in a sol state and transformed into a gel state to serve as drug reservoirs. Since the publication of the first paper on thermoresponsive injectable hydrogels as drug reservoirs, various types of thermoresponsive sol–gel transition polymers that exhibit a sol-to-gel transition upon heating have been designed [2,10,11]. However, few studies have focused on upper critical solution temperature (UCST)-type thermoresponsive sol–gel transition polymers that undergo a drastic change from a sol state to a gel state upon cooling. Lodge et al. reported a UCST-type sol–gel transition of P(*N*-isopropylacrylamide)-*b*-PEG-*b*-P(*N*-isopropylacrylamide) (PNIPAAm-PEG-PNIPAAm) in an ionic liquid [12]. PNIPAAm is the most studied thermoresponsive polymer, and it undergoes an LCST-type phase transition in aqueous media [13]. PNIPAAm also exhibits a UCST-type phase transition in certain ionic liquids. A 1-ethyl-3-methylimidazolium bis(trifluoromethylsulfonyl)imide ([EMIM][TFSI]) solution containing 10 wt% PNIPAAm-*b*-PEG-*b*-PNIPAAm triblock copolymer was a viscous liquid at room temperature. Upon cooling below 17 °C, the PNIPAAm blocks became insoluble, forming intra- and intermolecular assemblies in [EMIM] [TFSI]. As a result, PNIPAAm-*b*-PEG-*b*-PNIPAAm forms a gel network composed of PNIPAAm assemblies bridged with a PEG chain. Although natural polymers, such as agar, collagen, and gelatin, exhibit reversible UCST-type sol–gel transitions in aqueous media, few studies on synthetic polymers that exhibit UCST-type sol–gel transitions in aqueous media have been reported. 

**Figure 1:**
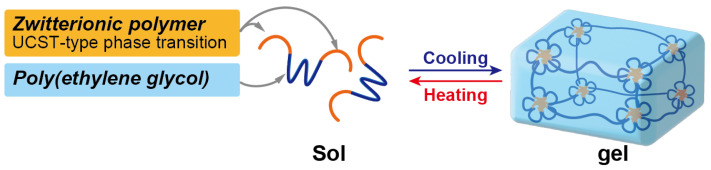
Schematic illustration of the thermoresponsive sol–gel transition of an ABA-type triblock copolymer containing zwitterionic polymer blocks that exhibit a UCST-type phase transition.

UCST-type phase transitions in aqueous media can be accomplished by introducing functional groups that exhibit strong intermolecular enthalpic interactions such as hydrogen bonding and dipole–dipole interactions [14,15]. For example, polymers exhibiting intermolecular hydrogen bonding, such as poly(acrylamide-*co*-acrylonitrile) (P(AAm-*co*-AN))-based copolymers [14,16,17], poly(*N*-acryloyl glycineamide) [18,19] and ureido-derivatized polymers [20,21,22], exhibit UCST-type thermoresponsive phase transitions. Some zwitterionic polymers also exhibit UCST-type phase transitions in aqueous media via intermolecular dipole–dipole interactions [23,24,25,26,27]. Zwitterionic polymers contain the same number of anionic and cationic groups within their repeating units. Zwitterionic polymers exhibit various fascinating properties such as high water solubility, low protein adsorption, and high lubrication. Therefore, zwitterionic polymers have been extensively studied for the development of biomaterials [28,29,30,31]. In addition, the high water solubility of zwitterionic polymers provides fascinating functional materials. For example, we designed a novel water-soluble emulsifier composed of highly water-soluble poly(2-methacryloyloxyethyl phosphorylcholine) (PMPC) and hydrophilic/lipophilic poly[oligo(ethylene glycol)methacrylate], which is different from conventional amphiphilic emulsifiers composed of hydrophilic and hydrophobic groups [32]. By utilizing a W/O emulsion stabilized with a double water-soluble emulsifier as a template for the polymerization reaction, stimuli-responsive soft materials, such as reductively responsive gel capsules and LCST-type thermoresponsive core–shell hydrogel particles, have been designed [32,33]. Zwitterionic polymers with sulfonate groups, such as sulfobetaine polymers with ammonium sulfonate groups [24,25] and sulfothetin polymers with sulfonium sulfonate groups [26], exhibit UCST-type thermoresponsive phase transitions. The UCST-type phase transition of these zwitterionic polymers is not affected by pH changes. Because methacrylates containing zwitterionic side chains allow for controlled radical polymerization, including atom transfer radical polymerization (ATRP) and reversible addition–fragmentation chain-transfer (RAFT) polymerization, zwitterionic polymers exhibiting a UCST-type thermoresponsive phase transition are useful platforms for designing UCST-type sol–gel transition polymers. However, UCST-type sol–gel transition has not yet been accomplished using ABA-type triblock copolymers with zwitterionic polymer blocks. In this study, we report the thermoresponsive sol–gel transition behavior of ABA-type triblock copolymers composed of UCST-type thermoresponsive zwitterionic polymer blocks and a PEG block (Figure 1). We investigated the effect of the structure of the ABA-type triblock copolymer, including the zwitterionic structure and chain length ratio between the zwitterionic polymer blocks and a PEG block, on the thermoresponsive sol–gel transition. To the best of our knowledge, this is the first report of an ABA-type triblock copolymer containing zwitterionic polymer blocks that exhibits UCST-type thermoresponsive sol–gel transitions under physiological ionic strength. Fundamental research on UCST-type thermoresponsive sol–gel transition polymers will contribute significantly to the development of smart biomaterials such as drug reservoirs and cell culture scaffolds.

## 2. Results and Discussion

### 2.1. Synthesis of Triblock Copolymer Containing Zwitterionic Polymer Blocks

**Scheme 1:**
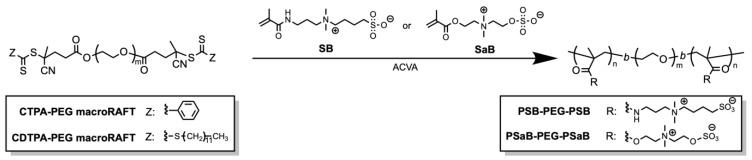
Synthesis of PSB-PEG-PSB and PSaB-PEG-PSaB triblock copolymers.

**Figure 2:**
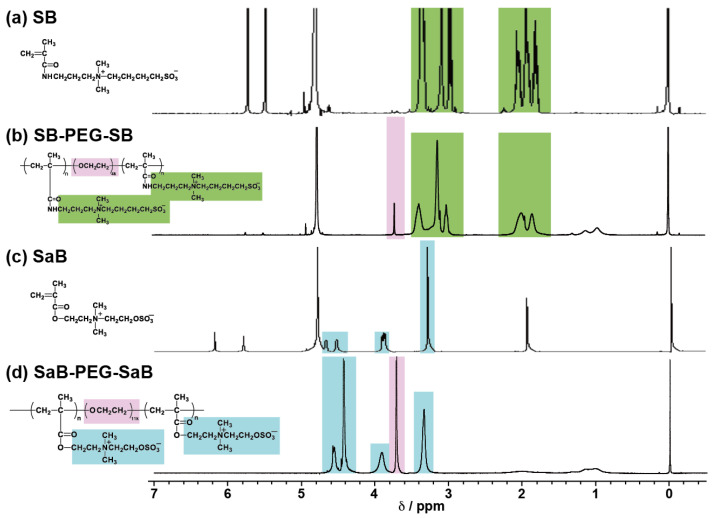
^1^H NMR spectra of (**a**) SB, (**b**) PSB_140_-PEG_91_-PSB_140_, (**c**) SaB, and (**d**) PSaB_97_-PEG_250_-PSaB_97_ (400 MHz, D_2_O containing 0.5 M NaNO_3_).

The ABA-type triblock copolymer composed of stimuli-responsive A blocks and water-soluble B block is the most promising candidate for designing stimuli-responsive sol–gel transition polymers. Although ABA-type triblock copolymers exhibiting LCST-type sol–gel transitions have been reported, there are few reports on UCST-type sol–gel transition polymers consisting of ABA-type triblock copolymers. The only example of an ABA-type sol–gel transition polymer with UCST-type thermoresponsive P(AAm-*co*-AN) blocks was reported by Fu and Zhao [34]. In this study, an ABA-type block copolymer composed of UCST-type thermoresponsive zwitterionic polymer blocks and a nonionic water-soluble PEG block was synthesized. Two types of zwitterionic polymers, ammonium sulfonate polymer (sulfobetaine polymer) and ammonium sulfate polymer (sulfabetaine polymer), were investigated for UCST-type sol–gel transition. The UCST of sulfobetaine and sulfabetaine polymers can be modulated by their chemical structures, such as the polymer backbone (methacrylate or methacrylamide), the spacer length between ammonium and sulfonate or sulfate, and the chain length of alkyl groups in the ammonium group [24,25,27]. In addition, the salt concentration affects the UCST of these polymers. The UCST of sulfobetaine polymers decreases drastically with increasing salt concentration, and this behavior disappears at physiological salt concentrations (i.e., 160 mM). Salts screen the intra- and intermolecular dipole–dipole interactions between the zwitterionic groups, resulting in the dissolution of sulfobetaine polymers at low temperatures [25,35]. On the other hand, sulfabetaine polymers exhibit UCST-type thermoresponsive behavior, even under physiological salt concentrations, owing to their strong intra- and intermolecular dipole–dipole interactions derived from the ammonium sulfate structure [36,37]. In this study, poly(4-((3-methacrylamidopropyl)dimethylammonio)butane-1-sulfonate) (PSB) and poly(3-((2-(methacryloyloxy)ethyl)dimethylammonio)ethylene-1-sulfate) (PSaB) were applied as UCST-type thermoresponsive blocks of an ABA-type triblock copolymer. PSB has a higher UCST than the standard sulfobetaine methacrylate polymer, poly[3-{(2-methacryloyloxyethyl) dimethylammonio}propane-1-sulfonate], and exhibits UCST-type thermoresponsive phase transition behavior in an aqueous solution containing <0.1 M of salt [24]. SB or SaB was polymerized from both ends of the PEG chain via RAFT polymerization using a PEG macroRAFT agent, as illustrated in Scheme 1. Figure 2 shows the ^1^H NMR spectra of PSB-PEG-PSB and PSaB-PEG-PSaB triblock copolymers. In the ^1^H NMR spectra of PSB-PEG-PSB and PSaB-PEG-PSaB, a peak assigned to the methylene protons of PEG was observed at 3.6–3.8 ppm (pink region). The broad peaks assigned to the methyl and methylene protons of SB (green region in Figure 2a,b) and SaB side chains (blue region in Figure 2c,d) were also observed. These results indicate that SB and SaB were successfully polymerized from the ends of the PEG macroRAFT agent. The synthesis of triblock copolymers was also confirmed by size exclusion chromatography (Figure S1). The degree of polymerization (*D*_p_) of PSB and PSaB was determined by integrating the methyl resonance of SB or SaB at approximately 3.3 ppm against the methylene proton of PEG at 3.6–3.8 ppm. The molecular weights and *D*_p_ of the triblock copolymers synthesized in this study are summarized in Table 1.

### 2.2. Thermoresponsive Behavior of Aqueous Triblock Copolymer Solutions under Dilute Conditions

**Figure 3:**
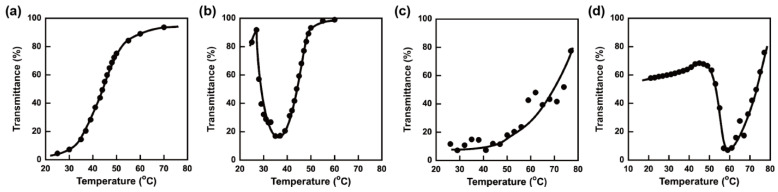
Changes in transmittance (800 nm) of aqueous PSB-PEG-PSB solutions ((**a**) PSB_140_-PEG_91_-PSB_140_; (**b**) PSB_96_-PEG_250_-PSB_96_) and PBS(−) containing PSaB-PEG-PSaB; ((**c**) PSaB_148_-PEG_91_-PSB_148_; (**d**) PSaB_97_-PEG_250_-PSaB_97_) as a function of temperature. The concentration of the triblock copolymer was 5 mg·mL^−1^. The solution was heated at a rate of 0.2 °C·min^−1^.

**Figure 4:**
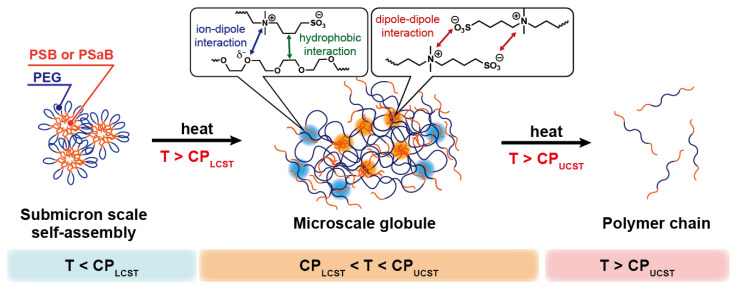
Schematic illustration of both the LCST- and UCST-like thermoresponsive phase transition behavior of PSB_96_-PEG_250_-PSB_96_ and PSaB_97_-PEG_250_-PSaB_97_ under dilute conditions.

The thermoresponsive behaviors of PSB-PEG-PSBs and PSaB-PEG-PSaBs were evaluated using the turbidity method. After dissolution at 80 °C, PSB-PEG-PSBs and PSaB-PEG-PSaBs were incubated at room temperature before turbidity measurements. PSB-PEG-PSBs dissolved completely in phosphate-buffered saline (PBS(−); *I* = 160 mM) at room temperature and exhibited no phase transition between 25 °C and 80 °C. The aqueous solution of PSB-PEG-PSB exhibited thermoresponsive phase transition. In contrast, PSaB-PEG-PSaBs exhibited a thermoresponsive phase transition in PBS(−) with high ionic strength. Figure 3a,b show the changes in the transmittance of the aqueous solutions containing PSB-PEG-PSBs as a function of temperature. PSB_140_-PEG_91_-PSB_140_ (the subscript number refers to the polymerization degree) formed insoluble aggregates in water at 25 °C because of the low water solubility of the long PSB blocks resulting from intermolecular complex formation by dipole–dipole interactions (Figure S2a). Upon heating, the transmittance increased drastically above 30 °C and reached approximately 90% at 70 °C. This result indicates that PSB_140_-PEG_91_-PSB_96_ exhibited UCST-type thermoresponsive behavior. Interestingly, PSB_96_-PEG_250_-PSB_96_, which has a longer hydrophilic PEG block and shorter PSB block than PSB_140_-PEG_91_-PSB_140_, exhibited both LCST- and UCST-like thermoresponsive phase transition behavior (Figure 3b). The transmittance of the aqueous PSB_96_-PEG_250_-PSB_96_ solution decreased significantly with an increasing temperature. Above 35 °C, the transmittance increased drastically and reached approximately 100% at 55 °C. PSaB-PEG-PSaBs in PBS(−) exhibited the same thermoresponsive phase transition behavior as that of the PSB-PEG-PSB system. The triblock copolymer with short PEG and long PSaB chains, PSaB_148_-PEG_91_-PSaB_148_, formed insoluble aggregates in PBS(−) at 25 °C. The transmittance of PBS(−) containing PSaB_148_-PEG_91_-PSaB_148_ gradually increased with an increasing temperature (Figure 3c). In contrast, PSaB_97_-PEG_250_-PSaB_97_, which has longer PEG blocks and shorter PSaB blocks than PSaB_148_-PEG_250_-PSaB_148_, formed submicron-scale self-assemblies at 25 °C (Figure S3). PSaB_97_-PEG_250_-PSaB_97_ exhibited both LCST- and UCST-like thermoresponsive behavior (Figure 3d). These results indicate that the chain length of PEG and the zwitterionic polymer affect the thermoresponsive phase transition behavior. PSB_140_-PEG_91_-PSB_140_ and PSaB_148_-PEG_91_-PSaB_148_, which had a short PEG block and long zwitterionic polymer blocks, exhibited only UCST-type thermoresponsive phase transition behavior. PSB_140_-PEG_91_-PSB_140_ and PSaB_148_-PEG_91_-PSaB_148_ precipitated at 25 °C because of the formation of insoluble aggregates by intermolecular dipole–dipole interactions of PSB or PSaB blocks. The increase in temperature induced the dissociation of intermolecular complexes of PSB_140_-PEG_91_-PSB_140_ or PSaB_148_-PEG_91_-PSaB_148_ owing to the shielding of the dipole–dipole interactions. Consequently, PSB_140_-PEG_91_-PSB_140_ and PSaB_148_-PEG_91_-PSaB_148_ became soluble at high temperatures. The aqueous PSB_96_-PEG_250_-PSB_96_ solution and PBS(−) containing PSaB_97_-PEG_250_-PSaB_97_ were turbid in appearance without obvious precipitation at 25 °C. DLS measurements revealed that PSB_96_-PEG_250_-PSB_96_ and PSaB_97_-PEG_250_-PSaB_97_ formed colloidally stable submicron-scale self-assemblies (Figure S2). The longer PEG chains of PSB_96_-PEG_250_-PSB_96_ and PSaB_97_-PEG_250_-PSaB_97_ were located on the surface of the water-insoluble core of PSB or PSaB self-assemblies to prevent aggregation and precipitation. Upon heating, the transmittance of the PSB_96_-PEG_250_-PSB_96_ and PSaB_97_-PEG_250_-PSaB_97_ solutions decreased significantly and then increased, showing both LCST- and UCST-like thermoresponsive behavior. The cloud and clearing points of triblock copolymer solutions are summarized in Table 2. The cloud point (CP_LCST_) was defined as the 50% transmittance of the triblock polymer solution in the LCST-type phase transition process. The clearing point (CP_UCST_) was defined as the 50% transmittance of the triblock copolymer solution in the UCST-type phase transition process. Few studies have reported that copolymers composed of nonionic and zwitterionic polymers exhibit unusual simultaneous LCST- and UCST-like phase transition behaviors. Laschewsky et al. reported that the block copolymer composed of linear PEG and PSB exhibited both LCST- and UCST-like phase transitions [38]. Morimoto and Yamamoto also reported that the random copolymer composed of poly(ethylene glycol)methacrylate and sulfabetaine methacrylate exhibited both LCST- and UCST-like phase transitions [39]. Although an obvious mechanism has not been established, both the LCST- and UCST-like thermoresponsive phase transitions seem to be induced by a combination of dipole–dipole, ion–dipole, and hydrophobic interactions. PSB and PSaB exhibited intra- and intermolecular dipole–dipole interactions. PSB-PEG and PSaB-PEG interactions arose from ion–dipole and hydrophobic interactions. The ammonium cation of PSB or PSaB interacted with the ether oxygen of PEG via ion–dipole interactions; the carbon chains in PSB or PSaB interacted with the ethylene moiety of PEG via hydrophobic interactions; and the PEG-PSB and PEG-PSaB interactions were sufficiently effective when the sizes of the blocks were similar. In this study, PSB_96_-PEG_250_-PSB_96_ and PSaB_97_-PEG_250_-PSaB_97_ exhibited both LCST- and UCST-like thermoresponsive phase transitions. The total *D*_p_ of PSB and PSaB blocks were nearly comparable to that of PEG (i.e., total *D*_p_ of PSB: 192; total *D*_p_ of PSaB: 194; *D*_p_ of PEG: 250). In contrast, PSB_140_-PEG_91_-PSB_140_ and PSaB_148_-PEG_91_-PSaB_148_, which have an approximately three-times greater total *D*_p_ of the zwitterionic polymer than that of PEG, exhibited only UCST-type thermoresponsive phase transition behavior. From these results, both the LCST- and UCST-like thermoresponsive phase transitions of PSB_96_-PEG_250_-PSB_96_ and PSaB_97_-PEG_250_-PSaB_97_ can be explained through the tentative model schematically illustrated in Figure 4. At room temperature, colloidally stable submicron-scale self-assemblies with PEG chains on their surfaces were formed by PSB-PSB or PSaB-PSaB intermolecular dipole–dipole interactions. An increase in temperature induced the shielding of dipole–dipole interactions. The partly dissociated PSB and PSaB chains interacted with the PEG chain via ion–dipole and hydrophobic interactions to form microscale globules. Further heating induced the shielding of the ion–dipole, dipole–dipole, and hydrophobic interactions, resulting in the dissociation of microscale globules. As a result, PSB_96_-PEG_250_-PSB_96_ and PSaB_97_-PEG_250_-PSaB_97_ showed both LCST- and UCST-like thermoresponsive phase transition behavior.

### 2.3. Thermoresponsive Behavior of Triblock Copolymers under Concentrated Conditions

**Figure 5:**
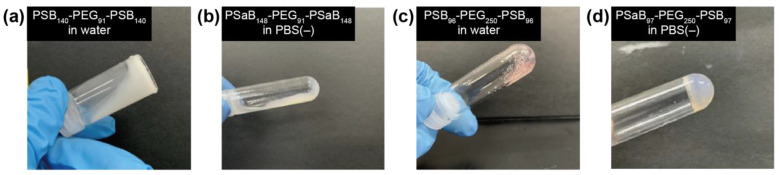
Photographs of (**a**) PSB_148_-PEG_91_-PSB_148_ in water (35 wt%), (**b**) PSaB_148_-PEG_91_-PSB_148_ in PBS(−) (15 wt%), (**c**) PSB_96_-PEG_250_-PSB_96_ (30 wt%) in water, and (**d**) PSaB_97_-PEG_250_-PSaB_97_ in PBS(−) (15 wt%) at 25 °C. The samples were prepared by dissolving the triblock copolymers at 80 °C, followed by cooling to room temperature.

The sol–gel transition of a stimuli-responsive ABA-type triblock copolymer depends on the polymer concentration. An increase in the triblock copolymer concentration induced network formation by bridging the polymer self-assemblies. In this study, the thermoresponsive sol–gel transition of a triblock copolymer was investigated at high concentrations. Figure 5 shows the photographs of PSB-PEG-PSBs in water and PSaB-PEG-PSaBs in PBS(−) after dissolution at 80 °C, followed by cooling to 25 °C. The PSB_140_-PEG_91_-PSB_140_ and PSaB_148_-PEG_91_-PSaB_148_ systems were watery turbid liquids with obvious precipitation of the triblock copolymers (Figure 5a,b). These results indicate that PSB_140_-PEG_91_-PSB_140_ and PSaB_148_-PEG_91_-PSaB_148_ did not exhibit thermoresponsive sol–gel transitions. Because the PSB and PSaB blocks of these triblock copolymers were much longer than the PEG block, the triblock copolymer was water-insoluble below the phase transition temperature of the PSB and PSaB blocks. This is the reason why the sol–gel transition was not observed.

**Figure 6:**
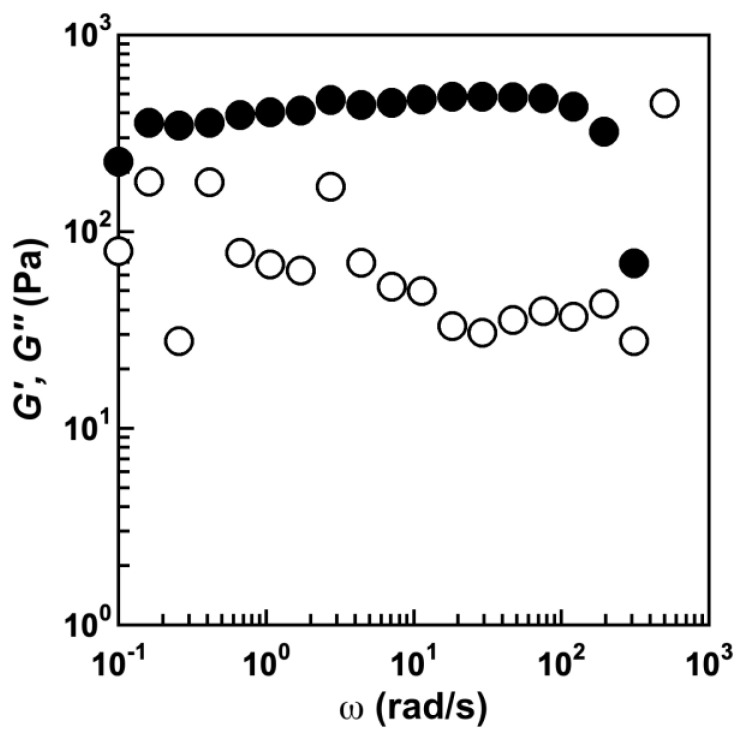
Changes in elastic (G’: ●) and loss moduli (G”: ○) of PSaB_97_-PEG_250_-PSB_97_ in PBS(−) as a function of angular frequency (ω).

**Figure 7:**
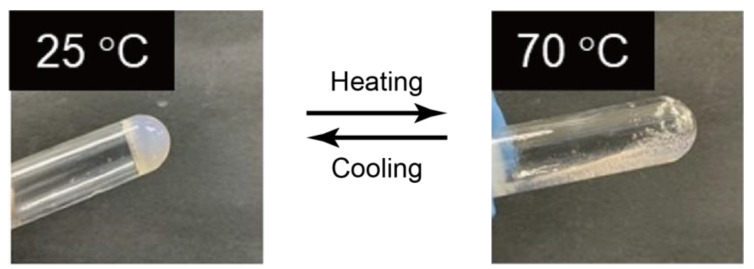
Photographs of the reversible sol–gel transition of PSaB_97_-PEG_250_-PSB_97_ in PBS(−). The PSaB_97_-PEG_250_-PSaB_97_ concentration in PBS(−) was 15 wt%.

In contrast, aqueous PSB_96_-PEG_250_-PSB_96_ solution was a viscous liquid without any precipitates at 25 °C (Figure 5c). The viscosity of the PSB_96_-PEG_250_-PSB_96_ solution increased with an increasing triblock copolymer concentration (Figure S4). Under dilute conditions, PSB_96_-PEG_250_-PSB_96_ formed self-assemblies with a diameter of approximately 100 nm (Figure S2b). An increase in the concentration of PSB_96_-PEG_250_-PSB_96_ induced bridge formation between the PSB_96_-PEG_250_-PSB_96_ self-assemblies, resulting in an increase in the apparent molecular weight. As a result, the viscosity of the PSB_96_-PEG_250_-PSB_96_ solution increased with an increasing concentration. However, a three-dimensional cross-linked network was not formed in the PSB_96_-PEG_250_-PSB_96_ solution. In contrast, when PSaB_97_-PEG_250_-PSaB_97_ was dissolved in PBS(−) at 80 °C and cooled to 25 °C, the solution exhibited a solid-like behavior (Figure 5d). Rheological measurements of the PBS(−) of PSaB_97_-PEG_250_-PSaB_97_ were performed at 25 °C, which is lower than its UCST (Figure 6). The storage modulus (G’) of the PBS(−) of PSaB_97_-PEG_250_-PSaB_97_ was larger than the loss modulus (G”) and was nearly independent of the angular frequency between 0.1 to 200 rad/s. The larger G’ value than *G*” value means that the PBS(−) of PSaB_97_-PEG_250_-PSaB_97_ undergoes gelation and behaves like a solid at 25 °C. In addition, when the resulting PSaB_97_-PEG_250_-PSaB_97_ gel was heated to 70 °C, a gel-to-sol transition was observed (Figure 7). An increase in temperature induced a dissociation of the PSaB_97_-PEG_250_-PSaB_97_ self-assemblies acting as physical crosslinks by the shielding of dipole–dipole interactions between PSaB blocks. As a result, the PSaB_97_-PEG_250_-PSaB_97_ gel underwent a phase transition to a sol state. Even though the chain length of PSB in PSB_96_-PEG_250_-PSB_96_ was almost the same as that of PSaB in PSaB_97_-PEG_250_-PSaB_97_, PSB_96_-PEG_250_-PSB_96_ did not exhibit a sol–gel transition but only increased in viscosity. PSaB with an ammonium sulfate side chain exhibited stronger intermolecular dipole–dipole interactions than PSB with an ammonium sulfonate side chain. These results imply that the strength of the dipole–dipole interaction is a key factor in designing UCST-type thermoresponsive sol–gel transition polymers with zwitterionic polymer blocks.

**Figure 8:**
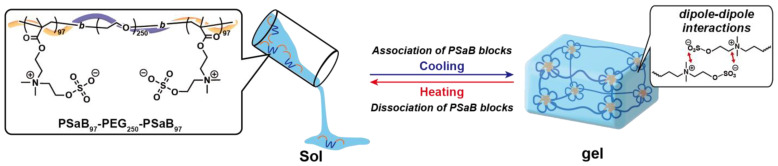
Schematic illustration of the UCST-type thermoresponsive sol–gel transition of the PBS(−) solution containing PSaB_97_-PEG_250_-PSaB_97_.

We conclude from these results that PSaB_97_-PEG_250_-PSaB_97_ is a reversible UCST-type thermoresponsive sol–gel phase transition polymer. The thermoresponsive sol–gel transition of PSaB_97_-PEG_250_-PSaB_97_ can be explained using the tentative model schematically illustrated in Figure 8. PSaB_97_-PEG_250_-PSaB_97_ was dissolved in PBS(−) above the phase transition temperature of the PSaB block. Upon cooling, the PSaB blocks of PSaB_97_-PEG_250_-PSaB_97_ were assembled by a strong dipole–dipole interaction to form water-insoluble PSaB self-assemblies bridged with hydrophilic PEG chains. Consequently, the PBS(−) solution containing PSaB_97_-PEG_250_-PSaB_97_ underwent a sol-to-gel transition. Heating of the resulting PSaB_97_-PEG_250_-PSaB_97_ gel induced the dissociation of PSaB_97_-PEG_250_-PSaB_97_ self-assemblies, acting as physical cross-links owing to the shielding of dipole–dipole interactions between the PSaB blocks. As a result, the PSaB_97_-PEG_250_-PSaB_97_ gel exhibited gel-to-sol transition. UCST-type thermoresponsive sol–gel transition polymers have potential applications as smart biomaterials such as cell culture scaffolds and drug reservoirs. Detailed studies on thermoresponsive sol–gel transition behavior are in progress, focusing on various aspects, including sol–gel phase diagrams, rheological studies, and the effects of the molecular weight and composition of the triblock copolymer on the sol–gel transition.

## 3. Conclusions

ABA-type triblock copolymers with UCST-type thermoresponsive zwitterionic polymer blocks were synthesized via RAFT polymerization using a PEG macroRAFT agent. Under dilute conditions, PSB_148_-PEG_91_-PSB_148_ and PSaB_148_-PEG_91_-PSB_148_, which had long zwitterionic polymer blocks and a short PEG block, exhibited UCST-type thermoresponsive behavior. On the other hand, PSB_96_-PEG_250_-PSB_96_ and PSaB_97_-PEG_250_-PSaB_97_ showed both LCST- and UCST-like phase transition behaviors because of the cooperative effect of the dipole–dipole interactions of PSB-PSB or PSaB-PSaB and the ion–dipole and hydrophobic interactions of PSB-PEG or PSaB-PEG. Under concentrated conditions, PSB_148_-PEG_91_-PSB_148_ and PSaB_148_-PEG_91_-PSB_148_ precipitated from the solution upon cooling and did not undergo a sol-to-gel transition. Because the PSB and PSaB blocks of these triblock copolymers were much longer than the PEG block, the triblock copolymer formed water-insoluble assemblies below UCST of the PSB and PSaB blocks. When the PSB_96_-PEG_250_-PSB_96_ solution was cooled from 80 to 25 °C, an increase in its viscosity was observed. In contrast, PSaB_97_-PEG_250_-PSaB_97_ underwent a drastic change from the sol state to the gel state upon cooling. These results imply that the strength of the dipole–dipole interaction is a key factor for UCST-type thermoresponsive sol–gel transition using zwitterionic polymers. Detailed studies on the sol–gel transition of PSaB-PEG-PSaB triblock copolymers are under investigation, with a focus on aspects such as polymer composition, phase diagrams, and rheological measurements. Although further research to determine the key factor for sol–gel transition of the PSaB-PEG-PSaB is necessary, it is likely to become an important material for constructing injectable drug reservoirs and cell culture scaffolds.

## 4. Materials and Methods

### 4.1. Chemicals

Poly(ethylene glycol)s (PEG; *M*_n_: 3900 and 11200) were kindly provided by NOF Corporation (Tokyo, Japan). 4-Cyano-4-(thiobenzoylthio)pentanoic acid (CTPA) and 4-cyano-4-[(dodecylsulfanylthiocarbonyl)sulfanyl]pentanoic acid (CDTPA) were purchased from Millipore Sigma (St. Louis, MO, USA). Dicyclohexylcarbodiimide (DCC), *N,N*-dimethylaminopyridine (DMAP), 2,2,2-trifluoroethanol (TFE), 1,4-butanesultone, 1,3,2-dioxathiane-2,2-dioxide, and *N,N*-dimethylaminopropyl methacrylate were purchased from Tokyo Chemical Industry Co., Ltd. (Tokyo, Japan). 4,4’-Azobis (4-cyanovaleric acid) (ACVA) and *N,N*-dimethylaminoethyl methacrylate were purchased from FUJIFILM Wako Pure Chemical Corporation (Osaka, Japan). Phosphate-buffered saline (PBS(−)) was prepared by dissolving NaCl (4.0 g), KCl, (0.10 g), Na_2_HPO_4_·12H_2_O (1.5 g), and KH_2_PO_4_ (0.10 g) into 500 mL of ultrapure water (Milli-Q, 18.2 MΩ·cm). All aqueous solutions were prepared using ultrapure water (Milli-Q, 18.2 MΩ·cm). Other solvents were obtained from commercial sources and used without purification.

### 4.2. Synthesis of PEG macroRAFT Agents

PEG, RAFT agent (CTPA or CDTPA), DCC, and DMAP were dissolved in dichloromethane (DCM), followed by stirring for 24 h. After the removal of the produced solid by filtration, the product was precipitated in diethyl ether. The isolated product was dried under vacuum to obtain PEG macroRAFT agents. The synthetic conditions are summarized in Table 3.

### 4.3. Synthesis of 4-((3-methacrylamidopropyl)dimethylammonio) butane-1-sulfonate (SB)

SB was synthesized by a previously reported method [24]. An amount of 4.0 mL of acetonitrile solution containing 1,4-butanesultone (5.44 g, 0.04 mol) was added dropwise to 4.0 mL of acetonitrile solution containing *N,N*-dimethylaminopropyl methacrylamide (3.40 g, 0.02 mol) for 30 min and then the mixture was stirred at room temperature for 48 h. The produced white solid was collected by filtration and washed with acetonitrile (20 mL). The product was then dried under vacuum to obtain SB as a white solid (4.59 g, 75% yield).

### 4.4. Synthesis of 3-{[2-(methacryloyloxy)ethyl]dimethylammonio}ethylene-1-sulfate (SaB)

SaB was synthesized by a previously reported method [38]. An amount of 32 mL of acetonitrile solution containing 1,3,2-dioxathiane-2,2-dioxide (6.20 g, 0.050 mol) was added dropwise to 32 mL acetonitrile solution containing *N,N*-dimethylaminoethyl methacrylate (8.95 g, 0.055 mol) for 30 min and then the mixture was stirred at room temperature for 48 h. The produced white solid was collected by filtration and washed with acetonitrile (20 mL). The product was then dried under vacuum to obtain SaB as a white solid (12.3 g, 87% yield).

### 4.5. Synthesis of Triblock Copolymers

Zwitterionic monomer (SB or SaB), PEG macroRAFT agents, and ACVA (0.27 mg, 1.0 μmol) were dissolved in TFE. The solution was deoxygenated by three freeze–pump–thaw cycles, followed by stirring at 90 °C under an Ar atmosphere. The PSB-PEG-PSBs were purified as follows: the polymer product was precipitated using methanol. The isolated product was then dried under vacuum to obtain the PSB-PEG-PSB triblock copolymer. PSaB-PEG-PSaBs were purified as follows: the reaction solution was poured into a seamless cellulose tubing (molecular cut-off: 12–14 kDa) and dialyzed against 1.0 M sodium chloride solution and ultrapure water. The solvent was removed by freeze-drying to obtain PSaB-PEG-PSaB triblock copolymer. The synthesis conditions are summarized in Table 4.

### 4.6. Phase Transition Temperature Measurements

A UV-Vis spectrophotometer (UV-1900i, Shimadzu Co., Kyoto, Japan) was used to evaluate the thermoresponsive phase transition of PSB-PEG-PSBs and PSaB-PEG-PSaBs by measuring their transmittance at 800 nm while heating the solutions at a constant rate of 0.2 °C·min^−1^. The cloud point (CP_LCST_) and clearing point (CP_UCST_) were defined as the 50% transmittance of the triblock polymer solution.

### 4.7. Rheological Measurement of the PSaB-PEG-PSaB Gel

PSaB_97_-PEG_250_-PSaB_97_ (100 mg) was added to 0.565 mL of ultrapure water or phosphate-buffered saline (PBS(−)) and was dissolved at 80 °C. The PSaB-PEG-PSaB solution was incubated at room temperature to obtain a PSaB-PEG-PSaB hydrogel. Rheological measurements of the PSaB_97_-PEG_250_-PSaB_97_ hydrogel were performed under a constant strain of 0.1% and angular frequency range of 0.1 to 500 rad/s at 25 °C using a Modular Compact rheometer MCR102 (Anton Paar GmbH, Graz, Austria).

## Figures and Tables

**Table 1:** Molecular weight and polymerization degree of triblock copolymers.

	PEG Block		Zwitterionic Polymer Block		Triblock Copolymer
	*M*_n_ (×10^3^) ^a)^	*D*_p_ ^a)^	*M*_n_ (×10^3^) ^b)^	*D*_p_ ^b)^	*M*_n_ (×10^3^) ^b)^
PSB_140_-PEG_91_-PSB_140_	3.9	91	42.9	140	89.8
PSB_96_-PEG_250_-PSB_96_	11.2	250	29.4	96	69.8
PSaB_148_-PEG_91_-PSaB_148_	3.9	91	45.4	148	94.7
PSaB_97_-PEG_250_-PSaB_97_	11.2	250	29.7	97	70.4

^a)^ Calculated from GPC curves using near-monodisperse poly(ethylene glycol) calibration standards. ^b)^ Calculated from ^1^H NMR.

**Table 2:** CP_LCST_ and CP_UCST_ of triblock copolymers in water and PBS(−).

	In Water		In PBS(−)	
	CP_LCST_ (°C)	CP_UCST_ (°C)	CP_LCST_ (°C)	CP_UCST_ (°C)
PSB_140_-PEG_91_-PSB_140_	–	43.2	n.d. ^a)^	n.d. ^a)^
PSB_96_-PEG_250_-PSB_96_	29.4	44.0	n.d. ^a)^	n.d. ^a)^
PSaB_148_-PEG_91_-PSaB_148_	n.d. ^b)^	n.d. ^b)^	–	73.8
PSaB_97_-PEG_250_-PSaB_97_	n.d. ^a)^	n.d. ^a)^	53.4	73.1

^a)^ Completely dissolved between 25 and 80 °C; ^b)^ insoluble aggregation was formed between 25 and 80 °C.

**Table 3:** Synthetic conditions of PEG macroRAFT agents.

	*D*_p_ of PEG	PEG	RAFT Agent		DCC	DMAP	DCM	Temp.
			CTPA	CDTPA				
CDTPA-PEG macroRAFT	91	3.00 g (0.77 mmol)		0.49 g (1.2 mmol)	0.32 g (1.5 mmol)	0.04 g (0.32 mmol)	25 mL	r.t.
CTPA-PEG macroRAFT	250	2.50 g (0.22 mmol)	0.28 g (1.0 mmol)		0.21 g (1.0 mmol)	0.02 g (0.16 mmol)	13 mL	40 °C

**Table 4:** Synthetic conditions of triblock copolymers.

	Zwitterionic Monomer		macroRAFT Agent		ACVA	TFE	Time
	SB	SaB					
PSB_140_-PEG_91_-PSB_140_	2.16 g (7.0 mmol)	–	0.12 g (25 μmol)	(CDTPA-PEG_91_ macroRAFT)	2.0 mg (7.1 μmol)	3.6 mL	8 h
PSB_96_-PEG_250_-PSB_96_	0.58 g (1.9 mmol)	–	0.050 g (4.1 μmol)	(CTPA-PEG_250_ macroRAFT)	0.46 mg (1.6 μmol)	1.5 mL	8 h
PSaB_148_-PEG_91_-PSaB_148_	–	1.28 g (4.6 mmol)	0.30 g (64 μmol)	(CDTPA-PEG_91_ macroRAFT)	5.2 mg (18 μmol)	2.2 mL	6 h
PSaB_97_-PEG_250_-PSaB_97_	–	1.07 g (3.8 mmol)	0.20 g (16 μmol)	(CTPA-PEG_250_ macroRAFT)	2.1 mg (7.5 μmol)	3.0 mL	6 h

## Data Availability

All data and materials are available on request from the corresponding author. The data are not publicly available due to ongoing research using a part of the data.

## Supplementary Materials

The following supporting information can be downloaded at: https://www.mdpi.com/article/10.3390/gels10050288/s1, Figure S1: Molecular weight distribution of triblock copolymers; Figure S2: Size distributions of PSB_140_-PEG_91_-PSB_140_ and PSB_96_-PEG_250_-PSB_96_ at 25 °C; Figure S3: Size distribution of PSaB_97_-PEG_250_-PSB_97_ at 25 °C; Figure S4: Photographs of PSB_96_-PEG_250_-PSB_96_ in water after heating at 80 °C, followed by cooling to 25 °C.
