# Supplementary material for: UCST-Type Thermoresponsive Sol–Gel Transition Triblock Copolymer Containing Zwitterionic Polymer Blocks
Source: Gels. 2024 Apr 24;10(5):288. doi: 10.3390/gels10050288 (PMC11121674; doi:10.3390/gels10050288)
Supplement: Supplementary file 1 [file gels-10-00288-s001.zip › gels-2964412-supplementary.pdf]

Supporting Information

UCST-type Thermoresponsive Sol-Gel Transition Triblock Copolymer Containing Zwitterionic Polymer Blocks

Akifumi Kawamura[1,2], Ryogo Takahashi[1], Takashi Miyata*[1,2]*

[1]Department of Chemistry and Materials Engineering and [2]Organization for Research and Development of Innovative Science and Technology, Kansai University, 3-3-35, Yamate-cho, Suita, Osaka 564-8680, Japan.

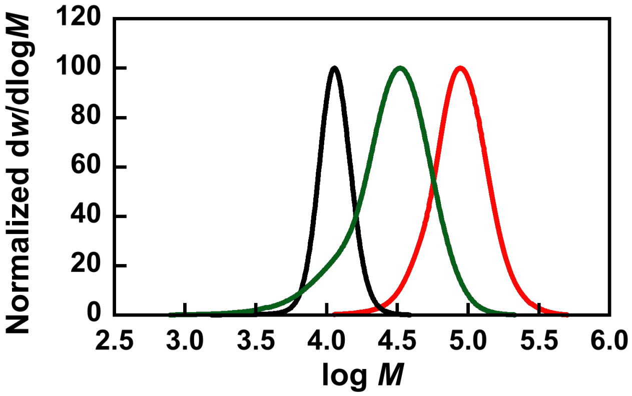

Figure S1. Molecular weight distributions (normalized to peak height) of PEG_{250} (black line), PSB_{96}-PEG_{250}-PSB_{96} (green line), and $PSaB_{97}$-PEG_{250}-$PSaB_{97}$ (red line). The size exclusion chromatography measurements were conducted using a Shimadzu Prominence HPLC system (Shimadzu corp., Kyoto, Japan) equipped with PFG 100 Å and PFG 1000 Å columns (Polymer Standards Service GmbH, Germany) at 40 °C under a flow rate of 1.0 mL/min using a refractive index detector. A 2,2,2-trifluoroethanol solution containing 20 mM sodium trifluoroacetate was used as an eluent. The molecular weight distributions were calculated using near-monodisperse poly(methyl methacrylate) standards.

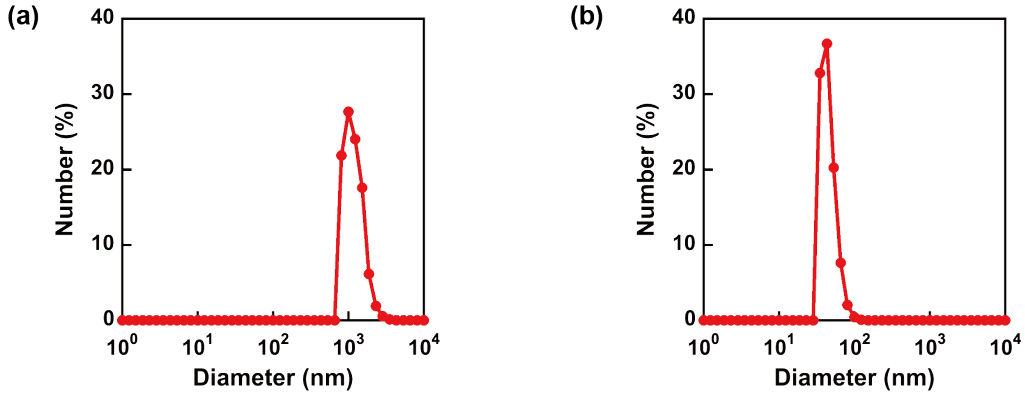

Figure S2. Size distributions of (a) PSB_{140}-PEG_{91}-PSB_{140} and (b) PSB_{96}-PEG_{250}-PSB_{96} at 25 °C. The concentration of triblock copolymers was 5 mg/mL. Size distributions of PSB-PEG-PSB self-assemblies were measured using ELS-Z1000 spectrometer (Otsuka Electronics Co., Ltd., Osaka, Japan) equipped with a He–Ne laser (λ = 633.8 nm) and backscatter optics. Size distribution was obtained using Marquart analysis.

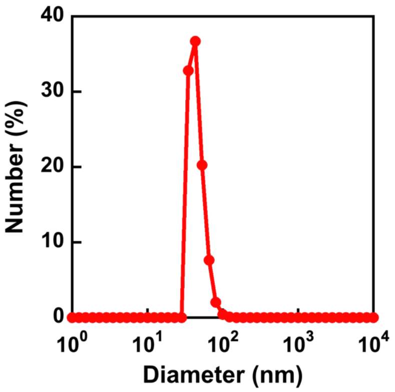

Figure S3. Size distribution of $PSaB_{97}$-PEG_{250}-$PSaB_{97}$ at 25 °C. The concentration of $PSaB_{97}$-PEG_{250}-$PSaB_{97}$ was 5 mg/mL. Size distributions of $PSaB_{97}$-PEG_{250}-PSB_{97} self-assemblies were measured using ELS-Z1000 spectrometer (Otsuka Electronics Co., Ltd., Osaka, Japan) equipped with a He–Ne laser (λ = 633.8 nm) and backscatter optics. Size distribution was obtained using Marquart analysis.

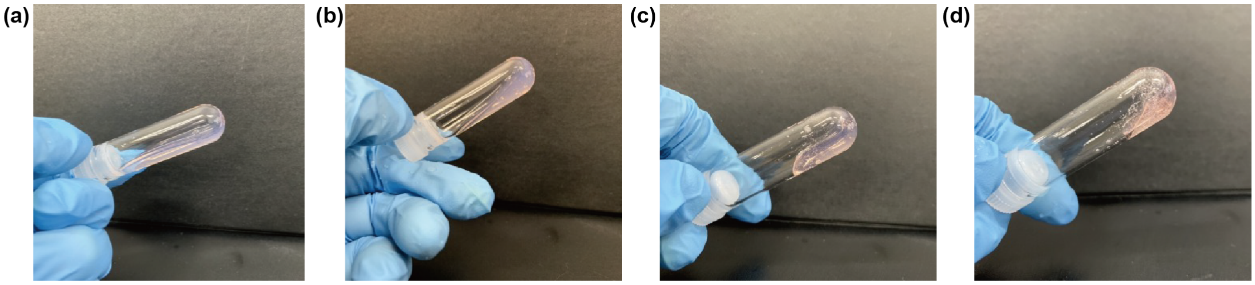

Figure S4. Photographs of PSB_{96}-PEG_{250}-PSB_{96} in water after heating at 80 °C, followed by cooling to 25 °C. The concentrations of PSB_{96}-PEG_{250}-PSB_{96} were (a) 15, (b) 20, (c) 25, and (d) 30 wt%.
